# Preservative solution that stabilizes erythrocyte morphology and leukocyte viability under ambient conditions

**DOI:** 10.1038/s41598-017-05978-7

**Published:** 2017-07-18

**Authors:** Rebecca D. Sandlin, Keith H. K. Wong, Leo Boneschansker, Thomas R. Carey, Kathleen L. Miller, Gregory Rose, Daniel A. Haber, Shyamala Maheswaran, Daniel Irimia, Shannon L. Stott, Mehmet Toner

**Affiliations:** 1 BioMEMS Resource Center, Center for Engineering in Medicine, & Department of Surgery, Massachusetts General Hospital, Harvard Medical School, Boston, MA 02114 USA; 2 Cancer Center & Department of Medicine, Massachusetts, MA General Hospital, Harvard Medical School, Boston, MA 02114 USA; 3 Howard Hughes Medical Institute, Chevy Chase, MD 20815 USA; 4 Cancer Center & Department of Surgery, Massachusetts General Hospital, Harvard Medical School, Boston, MA 02114 USA; 5 Cancer Center, Department of Medicine, & BioMEMS Resource Center, Center for Engineering in Medicine, Massachusetts General Hospital, Harvard Medical School, Boston, MA 02114 USA

## Abstract

The deterioration of whole blood *ex vivo* represents a logistical hurdle in clinical and research settings. Here, a cocktail preservative is described that stabilizes leukocyte viability and erythrocyte morphology in whole blood under ambient storage. Neutrophil biostabilization was explored using a sophisticated microfluidic assay to examine the effectiveness of caspase inhibition to stabilize purified neutrophils. Following 72 h ambient storage, neutrophils remained fully functional to migrate towards chemical cues and maintained their ability to undergo NETosis after stimulation. Furthermore, stored neutrophils exhibited improved CD45 biomarker retention and reduced apoptosis and mortality compared to untreated controls. To stabilize erythrocyte morphology, a preservative solution was formulated using Taguchi methods of experimental design, and combined with the caspase inhibitor to form a whole blood cocktail solution, CS_WB_. CS_WB_ was evaluated in blood from healthy donors and from women with metastatic breast cancer stored under ambient conditions for 72 h. CS_WB_-treated samples showed a significant improvement in erythrocyte morphology compared to untreated controls. Leukocytes in CS_WB_-treated blood exhibited significantly higher viability and CD45 biomarker retention compared to untreated controls. This 72 h shelf life under ambient conditions represents an opportunity to transport isolates or simply ease experimental timelines where blood degradation is problematic.

## Introduction

The emergence of cutting edge clinical and research technologies has created a significant demand for the biostabilization of blood during transportation and storage^1^. The inadequacy of current preservatives creates a logistical challenge for the dissemination of emerging blood-based analytical and diagnostic technologies that rely on viable, functional cells and where processing would more likely be performed at a centralized facility rather than locally, requiring short-term storage for transport. Specific applications range from functional immune cell assays^2^ to the culture of circulating tumor cells^3^. To mitigate storage-associated damage, cocktail preservative solutions have been developed for a range of tissues including organs, grafts and cell suspensions^4–8^. Development of a preservative to maintain viable blood cells is needed. Ideally the preservative would be compatible with ambient conditions, simplifying transport. Independence from continuous refrigeration would have further application for sample collection in low-resource settings. The ideal blood preservative should extend the shelf life of all cells in the blood for at least 72 h, sufficient time to transport samples globally and eliminate the need for immediate sample analysis. This timeframe would similarly enable sample storage over the weekend, easing experimental timelines.

Blood is a complex tissue containing an array of highly diverse cells, proteins and enzymes. This complexity complicates the development of blood preservatives. In particular, the short *ex vivo* half-life of neutrophils and their sensitivity to manipulation limits their use to ~2–4 h after collection^9^. Echinocytes, a spherical and spiculated form of erythrocyte, are frequently observed during blood storage and can form within 24 h after collection^10–12^. Echinocytes may interfere with microfluidic purification techniques for size-based blood cell sorting (Supplementary Table S1). This is due to an effective increase in echinocyte cell radius compared to discoid erythrocytes^13^. Current blood preservatives are either optimized for cold storage or rely on fixatives, resulting in loss of function and viability^14–18^. Typically in modern blood banking, whole blood is leukoreduced within 24 h, followed by refrigeration in a cold storage solution to delay cellular damage^15^. Several such preservatives have been developed including the FDA-licensed AS-1, AS-3 and AS-5 commercially available solutions^16, 17^. Component concentrations vary but typically consist of nutrients including NaCl, phosphate buffer system, adenine, dextrose and mannitol. As these additives have been selected specifically for erythrocytes, their formulations are not adequate for the storage of all blood components or ambient temperature, but serve as an excellent starting point for optimization. Potential supplements include apoptosis inhibitors to delay leukocyte deterioration and antioxidants to mitigate damage induced by production of oxidants, which has been reported for hypothermic blood storage^19–23^. Formulation optimization would then be necessary to stabilize whole blood under ambient storage. Statistical methods that enable the systematic evaluation of multiple parameters simultaneously have been developed for efficient experimental design. The Taguchi method for design of experiments is one such approach that has been used for a variety of applications in manufacturing and pharmaceutics^24, 25^. As opposed to full factorial design, the Taguchi method utilizes orthogonal arrays to test pair-wise combinations of each parameter, thereby reducing the number of experiments to a more practical number.

With the emergence of technologies for blood based diagnostics in cancer and sophisticated assays that rely on the molecular or physical features of viable cells, preservatives that enable cells to be stored and transported in their native state are urgently needed. Here, we describe a cost effective and easy to implement preservative that is capable of stabilizing erythrocyte morphology and leukocyte viability for 72 h at room temperature. As deterioration among leukocytes and erythrocytes occurs through distinct mechanisms, we first identified preservatives for each cell type independently. These preservative solutions were then combined and evaluated in whole blood. This whole blood cocktail solution, CS_WB_, was optimized for ambient storage conditions. To explore the applicability in patient care, CS_WB_ was tested in blood specimens from women with metastatic breast cancer. Unlike fixative solutions, CS_WB_ does not negatively impact cell viability, and may therefore have applications for molecular and functional assays that rely on blood cells. Thus, CS_WB_ has significant applications for medical and diagnostic technologies using peripheral blood.

## Materials and Methods

### Materials

Healthy donor blood was obtained from Research Blood Components, LLC (Brighton, MA) or from internal donors. Informed consent was obtained from all donors at MGH and experiments were approved by the MGH Institutional Review Board; all research was in accordance with approved guidelines. Blood specimens from patients with metastatic breast cancer were obtained after informed consent, per institutional review board protocol 05–300 at the Massachusetts General Hospital. All blood was collected in BD Vacutainer ACD Solution A collection tubes and used immediately after delivery.

Q-VD-OPh hydrate and Boc-D-FMK apoptosis inhibitors were obtained from APExBIO and Necrostatin-1 from Cayman Chemical. Each was dissolved in DMSO and stored at −20 °C. Additional blood additives were Certified ACS chemical grade and obtained from Fisher Scientific. Stock solutions were prepared weekly in sterile, ultrapure water. All data were analyzed using GraphPad Prism 6.

### Neutrophil isolation and storage

HetaSep sedimentation was used to separate leukocytes. Neutrophils were then isolated with the EasySep Human Neutrophil Enrichment Kit (STEMCELL Technologies, Vancouver, Canada) using the manufacturer’s protocol. Blood was processed immediately upon delivery and neutrophils were carefully handled using sterile technique throughout the isolation process.

Neutrophils were diluted with IMDM/20% FBS to a final concentration of 1 million cells/mL. 1 mL aliquots were then treated with 5 µM Q-VD-OPh, 50 µM Necrostatin-1, 50 µM Boc-D-FMK or vehicle control (1.25 µL DMSO) and purged with 5%CO_2_/5%O_2_. Separate tubes were used for each time point (24, 48, 72 or 96 h) and stored at 21 °C in a dark, temperature controlled room.

### Evaluation of neutrophil biomarker expression, viability, apoptosis and function

Following storage, neutrophils were centrifuged at 177 × g for 3 minutes. Supernatants were removed and neutrophils suspended in a 200 µL buffer solution containing 0.3% BSA, 10 mM CaCl_2_ and 10 mM HEPES. Neutrophils were stained with 1:100 Annexin-RPE and 1:500 SYTOX Green and stored in the dark for 15 minutes followed by treatment with 1:400 Hoechst and 1:100 CD45-APC for 15 minutes. Stained neutrophils were analyzed using an ImageStream^X^ Mk. II Imaging Flow Cytometer (Amnis Corporation). 30,000 events were collected for each sample. Data analysis was performed using the IDEAS software package. Focused, single, Hoechst positive events were gated from the cell population. Gates were applied to determine CD45 intensity, SYTOX and Annexin positive events based on the 0 h cell population for each matched dataset from the same donor.

Neutrophil migration was analyzed using a previously described microfluidic device^26^. Briefly, 3 layers of photoresist (SU8, Microchem, Newton, MA) were patterned on a silicon wafer, which was used as a mold to produce PDMS (Fisher Scientific, Fair Lawn, NJ) parts and bonded onto glass slides. The microfluidic network inside each device consisted of two arrays of up to 25 parallel channels (10 × 6 μm cross-section and 350 μm long) connected to both sides of one main channel with inlets and outlets. The devices were primed with a solution of fMLP (100 nM) and fibronectin (0.1% Sigma-Aldrich) for 15 min. The priming solution was then washed out, generating a chemokine gradient. Neutrophils were introduced into the main channel through tubing connected to the main inlet. The cells were gently flushed inside the main channel and trapped in cell capture traps to assure even loading of cells. Immediately after loading, cell migration was recorded using time-lapse imaging on a fully automated Nikon TiE microscope (10× magnification) with biochamber heated to 37 °C with 5% CO_2_, for 3 hours. The NIS Elements AR (v3.00) software package was used for data collection. Cell displacement was tracked manually using Image J.

NETosis was quantified by stimulating ~50,000 neutrophils for 12 hours with 50 nM Phorbol-12-myristate-13-acetate (PMA) (Sigma Aldrich, St. Louis, MO) inside a 24 well plate at 37 °C and 5%CO_2_. After NETs formation, cell media solution with 5 µM SYTOX Green was added to the wells, and used to stain the chromatin of the newly formed NETs.

### Optimization of CS-original for Erythrocyte Storage

The initial components selected for CS-original were derived from previously developed erythrocyte cold storage solutions^14–17^. CS-original optimization was accomplished using Taguchi methods^25^. An L_18_ mixed level orthogonal array was constructed where additives were tested at 2–3 concentrations (Table 1). Each additive was solubilized in sterile water and the HEPES stock solution was adjusted to pH 7.4 using sodium bicarbonate. The stock solutions were prepared at the following concentrations: 1 M HEPES, 10 mM adenine, 205 mM mannitol, 20mM N-acetyl-L-cysteine (NALC), 611 mM dextrose and 770 mM NaCl. Rather than test all 18 formulations simultaneously, sets of 6–13 conditions (and an untreated control) were selected for analysis for each patient sample due to limited blood volume. Each tube containing blood and preservative (1 mL volume) was purged with 5% CO_2_/5% O_2_ and stored at 21 °C in a dark room. At the indicated time, erythrocytes were rejuvenated by incubating with 2 mM adenosine (from 20 mM adenosine stock solution) in a 37 °C in a bead bath for 4 hours with intermittent mixing^27^. 10 µL blood was then diluted 1:100 in PBS, loaded onto a hemocytometer and imaged at 40x magnification using a Life Technologies EVOS FL or Nikon Eclipse 90i microscope. Approximately 300 erythrocytes were counted for each sample where echinocytes were identified by their distinct spiculations.

**Table 1:** Experimental conditions explored by Taguchi methods and resulting impact on erythrocytes.

Condition	HEPES (mM)	Adenine (mM)	Mannitol (mM)	NALC (mM)	Dextrose (mM)	NaCl (mM)	% discoid erythrocytes (76 h* storage) ± SEM
1	24	0.11	2.25	0.385	0	0	77.7 ± 16.4
2	24	0.11	4.5	0.77	7	8.5	84.6 ± 6.6
3	24	0.11	6.75	1.54	13.5	17	72.7 ± 10.7
4	24	0.22	2.25	0.385	7	8.5	85.0 ± 5.5
5**	24	0.22	4.5	0.77	13.5	17	78.6 ± 9.8
6	24	0.22	6.75	1.54	0	0	69.1 ± 15.0
7	24	0.44	2.25	0.77	0	17	86.8 ± 2.6
8	24	0.44	4.5	1.54	7	8.5	86.5 ± 5.1
9	24	0.44	6.75	0.385	13.5	8.5	74.5 ± 9.1
10	48	0.11	2.25	1.54	13.5	8.5	74.8 ± 10.5
11	48	0.11	4.5	0.385	0	17	83.3 ± 2.8
12	48	0.11	6.75	0.77	7	0	93.8 ± 3.8
13	48	0.22	2.25	0.77	13.5	0	86.0 ± 6.3
14	48	0.22	4.5	1.54	0	8.5	82.5 ± 6.2
15	48	0.22	6.75	0.385	7	17	72.3 ± 13.7
16	48	0.44	2.25	1.54	7	17	71.6 ± 17.1
17	48	0.44	4.5	0.385	13.5	0	78.5 ± 11.2
18***	48	0.44	6.75	0.77	0	8.5	94.7 ± 1.5
WB Control	—	—	—	—	—	—	69.3 ± 9.7

*following 72 h ambient storage, cells were incubated 4 h with adenosine (76 h total). **CS-Original formulation. ***CS_RBC_ formulation.

### Storage and characterization of erythrocytes and leukocytes from whole blood

The optimized erythrocyte preservative, CS_RBC_ (Taguchi formulation 18) was formulated as described and treated with Q-VD-OPh immediately before use. Blood was added to the CS_WB_ solution and purged with 5% CO_2_/5% O_2_ and stored at 21 °C in a dark, temperature controlled room (1 mL total volume). A control was prepared in an identical manner without preservatives. Samples were analyzed at 0 h and 72 h. For the 72 h samples, echinocytes in control (untreated) and CS_WB_-preserved blood were enumerated following the 4 h rejuvenation treatment with adenosine.

Leukocytes were isolated from whole blood prior to adenosine treatment by erythrocyte lysis using manufacturer’s recommended procedure using ammonium chloride solution (STEMCELL Technologies). Purified leukocytes were immediately stained and examined using flow cytometry as described previously. All leukocyte and erythrocyte data was paired except for Brx222 and Brx145. A summary of patient characteristics can be found in Supplementary Table S2.

## Results

### Characterization of erythrocyte and leukocyte degradation in whole blood under ambient storage conditions

**Figure 1:**
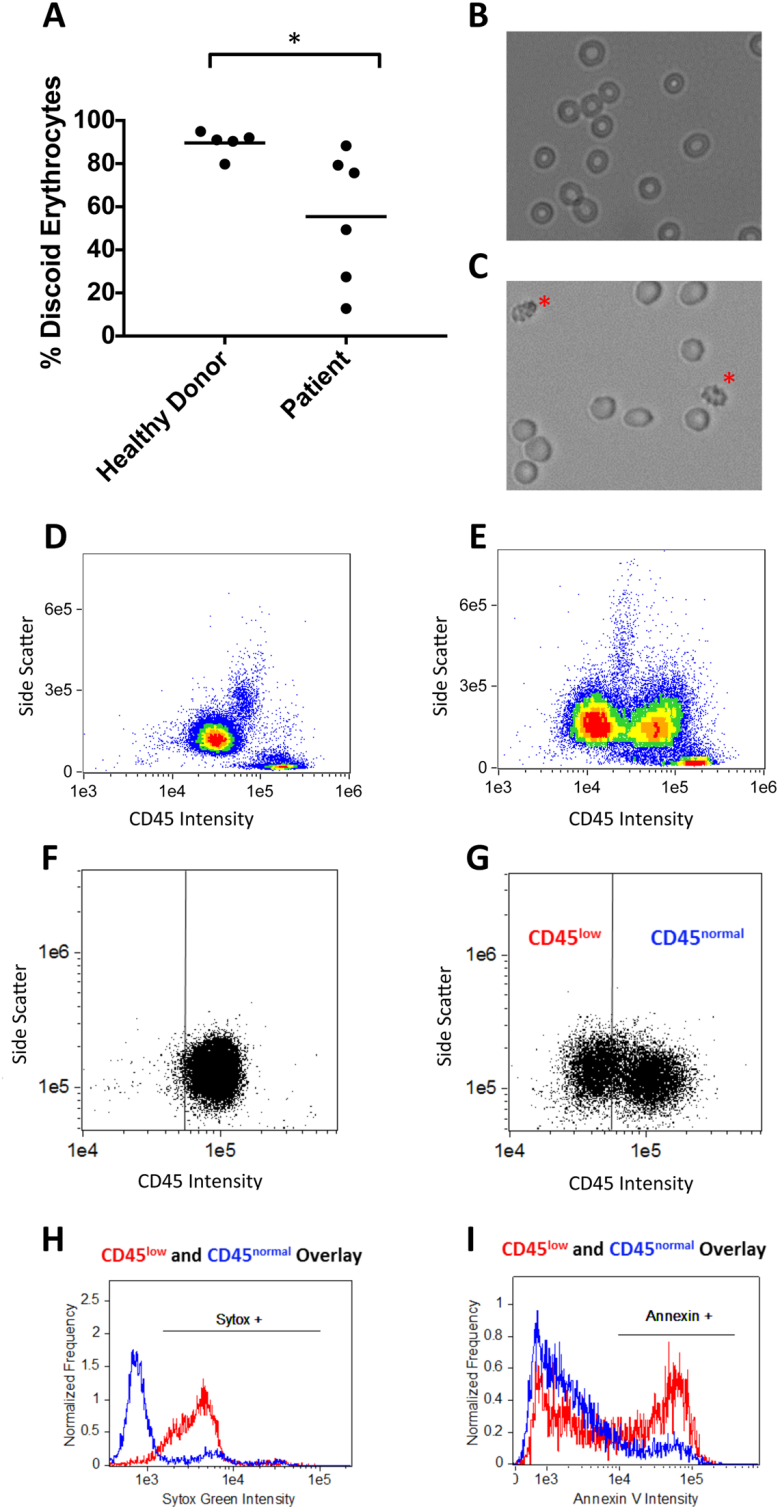
Degradation of whole blood components under ambient storage. Whole blood was collected from either healthy donors or metastatic cancer patients and stored under ambient conditions. Echinocytes were enumerated for each sample after 24 h storage. (**A**) Fewer echinocytes were observed in healthy donor samples (89.6 ± 5.8% discoid erythrocytes) compared to patient samples (55.5 ± 30.6% discoid erythrocytes, p < 0.05, t-test, values reported as mean ± standard deviation). (**B**) Images taken at 24 h show erythrocytes appear normal in the healthy donor blood sample compared to (**C**) image from patient sample where extensive echinocyte formation occurs (echinocytes noted with asterisk). (**D**) CD45 v Side Scatter plot reveals the expected distribution of leukocytes in a freshly collected blood sample compared to (**E**) the leukocytes collected from whole blood following 72 h ambient storage, where a new population of leukocytes with reduced CD45 expression was observed. (**F**–**I**) To study leukocyte degradation more closely in a simplified manner, neutrophils were isolated from fresh healthy donor blood and stored in culture media followed by analysis by flow cytometry. (**F**) CD45 expression in fresh neutrophils compared to (**G**) 72 h neutrophils shows this population undergoes extensive degradation. The CD45^low^ neutrophil population was shown to contain a greater number of (**H**) dead and (**I**) apoptotic cells compared to the CD45^normal^ population.

To develop a preservative for erythrocytes and leukocytes in whole blood, we first characterized their deterioration under ambient storage conditions. Erythrocyte quality was assessed based on echinocyte enumeration. Echinocytes are commonly observed in aged blood as spherical, spiculated cells compared to biconcave disc shaped erythrocytes^28^. Peripheral blood was collected from healthy donors and women with metastatic breast cancer and stored under ambient conditions. After 24 h, 89.6 ± 5.8% of the erythrocytes in the healthy donor samples exhibited normal discoid morphology compared to 55.5 ± 30.6% in patient samples (Fig. 1A–C). As each data point represents a unique donor, it is apparent that patient samples exhibit increased variability among individual donors compared to healthy donors.

CD45, a highly conserved glycoprotein expressed on the leukocyte membrane and routinely implemented for immunophenotyping by flow cytometry, was utilized here to establish storage-related deterioration. Figure 1D shows the density plot of fresh leukocytes from healthy donor blood compared to those stored for 72 h under ambient conditions (Fig. 1E). Over the course of 72 h storage, a population of cells forms that exhibits a significant reduction in CD45 intensity (CD45^low^). This CD45^low^ population is not present in fresh blood. Leukocytes from patient blood were also examined and found to similarly form a CD45^low^ cell population.

As neutrophils exhibit a short *ex vivo* half-life and account for approximately 45–70% of the total leukocyte population, we hypothesized that the CD45^low^ cell population consists primarily of damaged neutrophils. To validate this hypothesis, a simplified assay consisting of purified neutrophils in media was developed to quantify degradation during storage. Neutrophils isolated from fresh blood were observed over 96 h in 24 h intervals. The control condition of IMDM/20%FBS was selected due to improved neutrophil viability compared to serum-free media (Supplementary Figure S1). Figure 1F,G shows the density plot of fresh neutrophils compared to those stored under ambient conditions for 72 h, demonstrating the emergence of a CD45^low^ population over time. Histograms of SYTOX Green and Annexin V intensity were then generated to compare cell death and apoptosis among the CD45^normal^ and CD45^low^ populations. Figure 1H,I reveals that increased apoptosis and mortality is observed in the CD45^low^ population compared to the CD45^normal^ cells, suggesting a correlation between biomarker loss and apoptosis and death during sample storage.

### Caspase inhibitors delay apoptosis and death in purified neutrophils stored under ambient conditions for up to 96 h

As the CD45^low^ population correlated with increased apoptosis and death, caspase inhibition was evaluated as an approach to stabilize leukocytes during blood storage. Rather than test caspase inhibitors in whole blood, isolated neutrophils were examined as this is a simpler approach and these cells deteriorate quickly *ex vivo*. Q-VD-OPh and Boc-D-fmk are broad-spectrum caspsase inhibitors that have been used to investigate apoptotic pathways in neutrophils^29, 30^. A study by Wardle *et al*. demonstrated that caspase inhibition effectively blocks apoptosis in neutrophils and Q-VD-OPh in particular prevents apoptosis after 20 h at 37 °C^29^. Q-VD-OPh and Boc-D-fmk were also found to prevent Actinomycin-D induced apoptosis in WEHI-231 cells at a concentration of 5 µM and 50 µM, respectively^31^. Based on these data, we selected Q-VD-OPh and Boc-D-fmk for evaluation for leukocyte storage. To evaluate the role of non-apoptotic pathways in storage related leukocyte deterioration, a necroptosis inhibitor, Necrostatin-1, was evaluated at a 50 µM concentration, near that of published methods^32, 33^.

**Figure 2:**
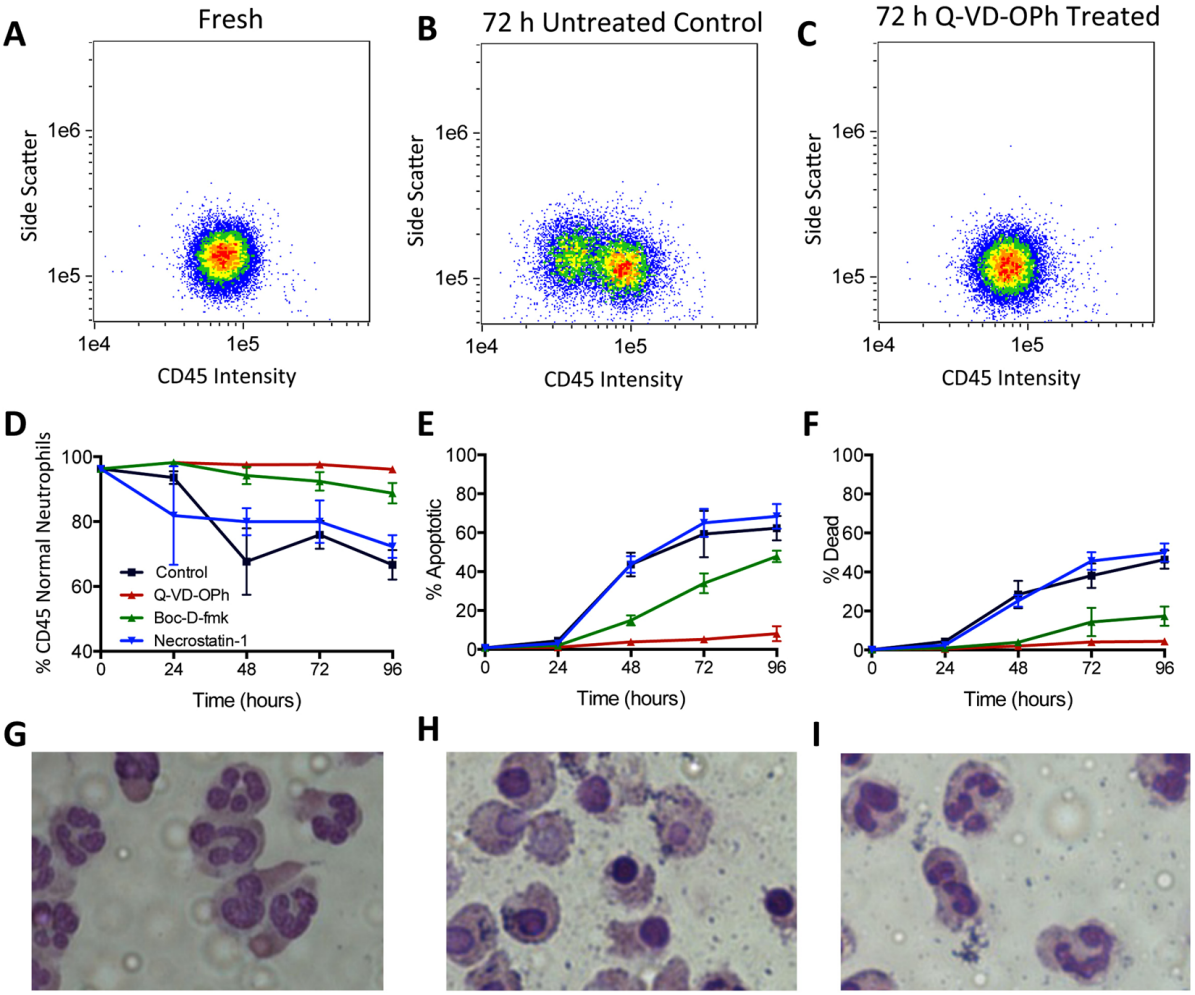
Evaluation of preservatives to stabilize neutrophils. (**A**) CD45 and side scatter of neutrophils isolated immediately after blood collection was determined using flow cytometry for comparison to those stored under ambient conditions in culture media using flow cytometry. (**B**) Neutrophils stored for 72 h without supplementation demonstrate CD45 degradation. (**C**) Neutrophils supplemented with the Q-VD-OPh apoptosis inhibitor exhibit normal CD45 expression levels following 72 h storage. Comparison of neutrophil (**D**) CD45 expression, (**E**) apoptosis and (**F**) viability over time in the presence of apoptosis or necrosis inhibitors (n = 4 independent healthy donors, plotted as average ± standard error). Representative image of isolated neutrophils taken (**G**) immediately after blood collection compared to those stored for 72 h (**H**) without supplementation or (**I**) supplemented with Q-VD-OPh shows improved nuclear morphology compared to the untreated control.

Figure 2 reveals the effects of each inhibitor on neutrophil biostabilization. Initially, 96.3 ± 0.8% of neutrophils are CD45^normal^, compared to only 66.7 ± 4.6% in the untreated control after 96 h. While Necrostatin-1 offered no significant improvement on CD45 expression compared to the untreated control (p > 0.05), both Boc-D-fmk and Q-VD-OPh apoptosis inhibitors dramatically improved CD45 stability where 88.8 ± 3.2% and 96.1 ± 1.0% of neutrophils were CD45^normal^ after 96 h, respectively. Similarly, Necrostatin-1 resulted in no significant improvement on viability and apoptosis compared to the untreated control where 46.4 ± 4.7% were dead and 62.3 ± 6.3% of cells were apoptotic (p > 0.05). In contrast, both Q-VD-OPh (4.5 ± 1.7%) and Boc-D-fmk (17.4 ± 4.9%) treated neutrophils showed significant improvement in viability after 96 h of storage compared to the untreated control (p < 0.001 and p < 0.01, respectively). Whereas only 8.0 ± 3.9% of Q-VD-OPh-treated neutrophils were apoptotic after 96 h, 47.8 ± 3.0% and 62.3 ± 6.3% of cells in the Boc-D-fmk and control were apoptotic, respectively. Further evaluation of cell morphology shows that neutrophils treated with apoptosis inhibitors exhibit normal nuclear morphology compared to the untreated control where a condensed nucleus is observed, suggesting apoptosis.

### Q-VD-OPh preserves neutrophil chemotaxis and NETosis in cells stored under ambient conditions for up to 72 hours

The aforementioned data demonstrates that caspase inhibition improves neutrophil viability when stored 96 h under ambient conditions. However, the functional integrity of neutrophils was still unclear. Therefore, we assessed neutrophil chemotaxis and NETosis to determine neutrophil functionality following storage. Q-VD-OPh was selected for this study based on superior performance.

**Figure 3:**
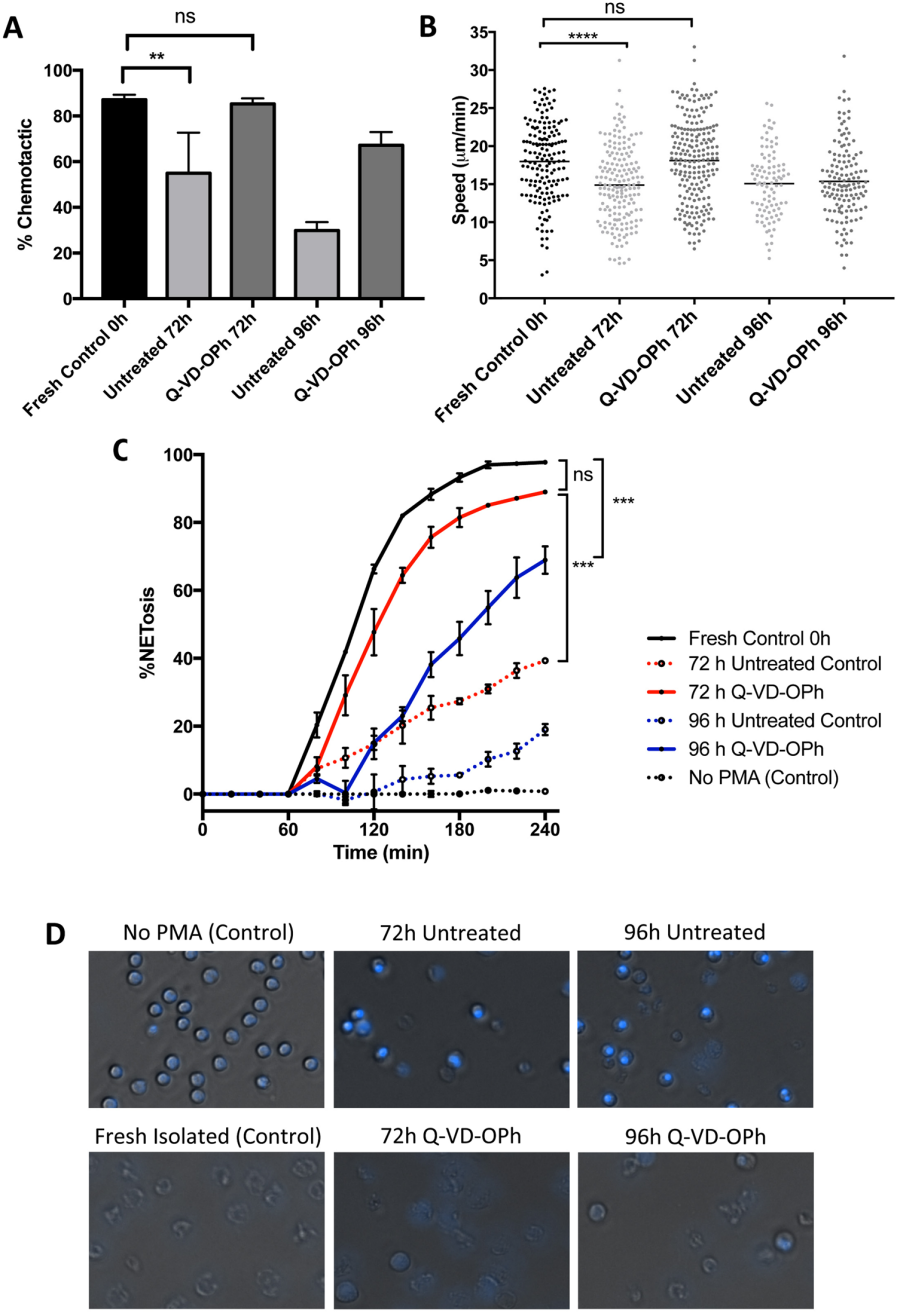
Ambient preservation of neutrophils. (**A**) Percent migration was established for isolated neutrophils following ambient storage (0 h v 72 h control, p < 0.01, t-test). (**B**) The migratory speed further shows that Q-VD-OPh treated cells appear indistinguishable from fresh neutrophils (0 h v 72 h control, p < 0.0001, t-test). (**C**) Following storage, the percentage of neutrophils that undergo NETosis over time in response to 10 nM PMA was quantified. Q-VD-OPh treated cells form more NETs compared to the untreated control (p < 0.001, Two-way ANOVA with Bonferroni multiple comparison post-test). The ability for Q-VD-OPh treated neutrophils to form NETs is not significantly impacted following 72 h ambient storage. (**D**) NETs formation after 240 min PMA stimulation in DAPI-stained neutrophils following 72–96 h ambient storage. All error bars represent standard deviation, n = 3.

Neutrophil chemotaxis was quantified using a microfluidic migration platform developed by Boneschansker, *et al*.^26^. Briefly, this device allows for the quantification of neutrophil chemotaxis in real-time at single cell resolution. Neutrophils were isolated from fresh blood and stored under ambient conditions for 72–96 h to determine the stabilizing effects of Q-VD-OPh compared to the untreated control. Functional integrity was evaluated based on the percentage of cells that undergo chemotaxis and their migratory speed. Figure 3A reveals that 87.1 ± 5.3% of freshly isolated neutrophils are chemotactic. After 72 h storage, 85.3 ± 6.6% of the Q-VD-OPh treated and 55.0 ± 17.7% of the untreated control cells migrated, suggesting that Q-VD-OPh serves as an excellent neutrophil preservative for ambient storage (Supplementary Videos S1, S2). However these values were greatly reduced following 96 h storage where only 67.2 ± 11.5% and 29.9 ± 7.3% of the Q-VD-OPh treated and control cells migrated, respectively. While Q-VD-OPh substantially extends the functional integrity of *ex vivo* neutrophils during storage, a limit of ~72 h is evident based upon their ability to perform chemotaxis.

Neutrophil function in preserved samples was further assessed based on migratory speed. Among the chemotactic neutrophils, fresh neutrophils exhibited an average speed of 18.0 µm/sec (Fig. 3B). Decreased speed was not observed following 72 h storage in neutrophils treated with Q-VD-OPh where the average speed was 18.1 µm/sec. In untreated cells, the average speed of those that still migrated fell to 14.9 µm/sec (p < 0.0001 compared to fresh control). The protective effect was lost after 96 h where the average speed was 15.4 and 15.1 µm/sec for the Q-VD-OPh treated sample and control, respectively (p < 0.0001 compared to fresh control).

Finally, NETosis was measured in preserved samples. Figure 3C,D demonstrates that Q-VD-OPh preserves NETosis following 72 h ambient storage where no significant reduction is observed compared to fresh control, though losses are observed after 96 h (p < 0.001). However, significant loss of NETs formation is observed in the untreated control as early as 72 h after storage (p < 0.001).

### Optimization of CS-Original for patient erythrocytes

In blood banking, the *ex vivo* half-life of packed erythrocytes is increased by cold storage solutions^16, 17^. Here, these additives were used as a starting point to formulate a preservative to stabilize erythrocyte morphology in whole blood stored under ambient conditions. In addition, HEPES buffer (pH adjusted to 7.4 using sodium bicarbonate) was used to stabilize pH and the antioxidant n-acetyl-l-cysteine (NALC) was used to minimize oxidative injury, which has been implicated in hypothermic storage-associated damage^19–23^.

The initial formulation, CS-original, was found to stabilize healthy donor erythrocytes for at least 96 h when combined with a post-storage incubation with 2 mM adenosine at 37 °C (Supplementary Figure S2). This formulation was not sufficient for blood specimens from women with metastatic breast cancer, where only 78.6 ± 9.8% of erythrocytes exhibited discoid morphology following a post-storage rejuvenation treatment with adenosine after 72 h of storage (Table 1, Formulation 5). To determine whether the additive components could be optimized for patient blood compatibility, Taguchi methods of experimental design were used to reduce the number of combinations to a manageable set^25^. Each formulation was added to patient blood (n = 5 unique donors) and treated with adenosine following 72 h ambient storage. Table 1 demonstrates that several formulations offer superior erythrocyte stabilization compared to the untreated control and CS-original. In particular, Taguchi formulation 18 (hereafter referred to as CS_RBC_) leads to the recovery of 94.7 ± 1.5% discoid erythrocytes.

### CS_WB_ improves leukocyte viability and biomarker expression and maintains erythrocyte morphology in healthy donor and patient whole blood stored at ambient temperature for 72 h

**Figure 4:**
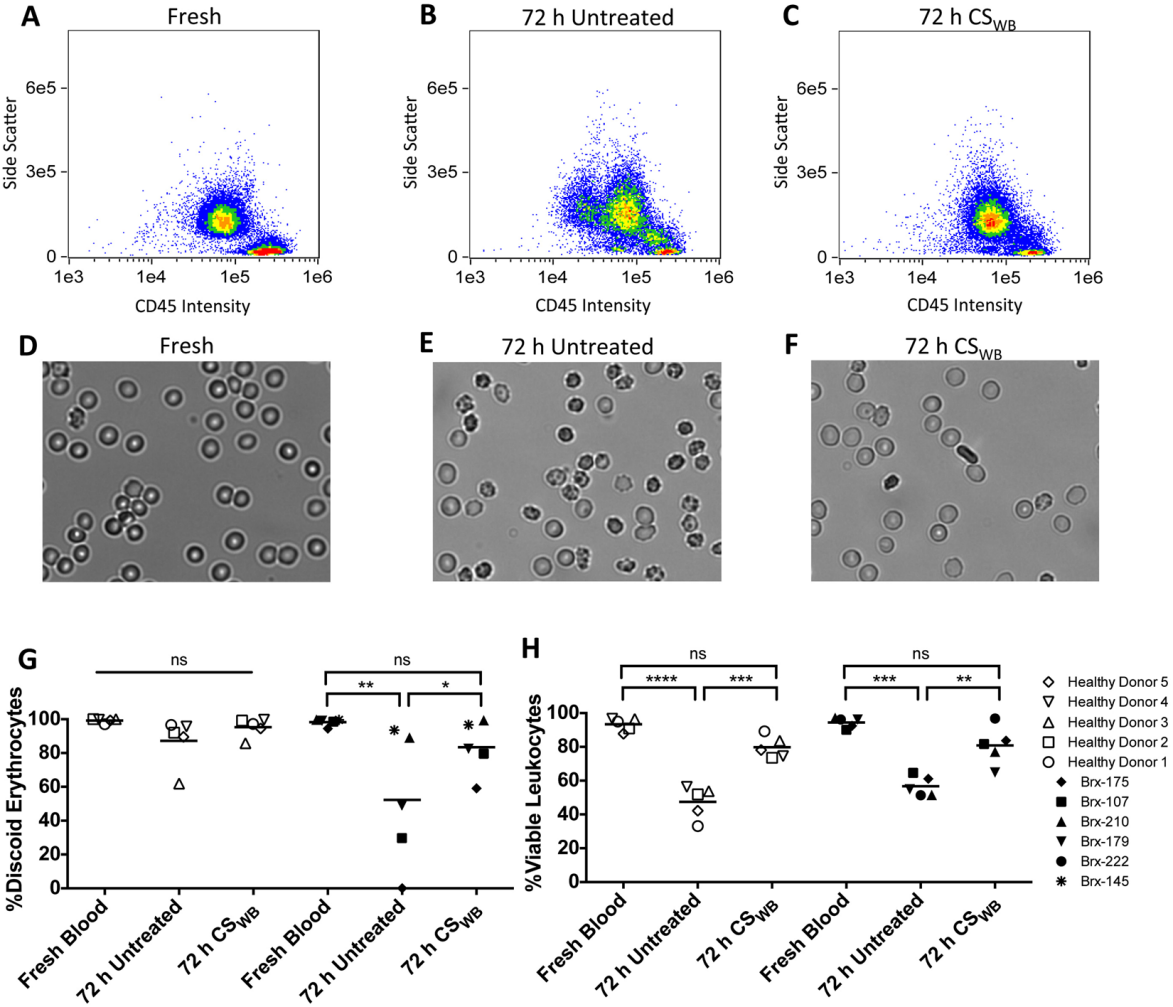
Stabilization of whole blood under ambient conditions. Leukocytes were isolated from whole blood and stained with CD45 and SYTOX Green then analyzed using flow cytometry. (**A**) CD45 v side scatter intensity was plotted for leukocytes obtained from fresh blood for comparison to those stored for 72 h that were either (**B**) untreated (whole blood only) or (**C**) treated with CS_WB_. In parallel, erythrocytes were examined for each condition including (**D**) fresh control, (**E**) untreated control and (**F**) CS_WB_-treated after 72 h storage. (**G**) The erythrocytes and (**H**) leukocytes of 5 healthy donors (open symbols) and 5 patients (Brx, closed symbols) were analyzed from fresh blood and following 72 h storage in samples that were CS_WB_-treated or untreated controls (n = 5, 1-way repeated measures ANOVA).

The data presented demonstrates that CS_RBC_ improves retention of erythrocyte discoid morphology and Q-VD-OPh increases neutrophil viability, biomarker retention and functional integrity. Since the additives were selected based upon homogeneous populations, we next investigated their combined effects for whole blood preservation at ambient temperature. CS_RBC_ and 5 µM Q-VD-OPh (hereafter referred to as CS_WB_) were added to freshly collected healthy donor or patient whole blood. After 72 h, cells were evaluated based upon erythrocyte morphology, leukocyte CD45 expression and viability. CD45/Side Scatter density plots in Fig. 4A–C reveal that the CS_WB_ treated sample exhibits improved CD45 stabilization compared to the untreated control where significant deterioration in CD45 intensity is observed. In fresh blood, 93.4 ± 3.9% (healthy donor) and 94.4 ± 3.0% (patient) of leukocytes were viable. After 72 h ambient storage in CS_WB_, 79.8 ± 6.6% and 80.8 ± 11.5% of the healthy donor and patient leukocytes were viable, respectively. This compares favorably to the control, where 47.9 ± 9.7% and 56.7 ± 5.9% of healthy donor and patient leukocytes were viable after storage. While the functional integrity of these leukocyte subpopulations was not characterized, treatment with CS_WB_ led to a significant improvement in viability.

In fresh blood, 99.2 ± 1.3% and 98.3 ± 2.2% of erythrocytes exhibit normal discoid morphology in healthy donor and patient samples, respectively (Fig. 4D–G). After 72 h storage followed by the adenosine rejuvination treatment, this is reduced to 95.3 ± 5.7% and 87.2 ± 14.4% in CS_WB_-treated and control blood among healthy donors. This reduction is more pronounced in patient samples where 83.5 ± 16.1% and 52.3 ± 40.0% of the CS_WB_- treated and control blood samples exhibit normal discoid morphology following 72 h ambient storage, respectively. Figure 4G reveals significant donor variability among patient samples stored for 72 h, which explains the high deviation observed.

## Discussion

As blood degrades rapidly *ex vivo*, preservatives are needed that stabilize whole blood under ambient storage, thereby enabling sufficient time to transport specimens to distant laboratories or processing facilities and ease diagnostic or experimental timelines. Here, we developed a cocktail preservative that i) preserves neutrophil function in homogenous cell suspensions ii) preserves leukocyte viability and CD45 biomarker expression in whole blood and iii) stabilizes erythrocyte morphology in whole blood. The preservative is compatible with ambient storage for up to 72 h.

Our initial studies demonstrated that CD45^low^ leukocytes in stored blood samples consist primarily of dead or apoptotic neutrophils, prompting the evaluation of caspase inhibitors as preservatives for ambient storage. Q-VD-OPh was found to stabilize leukocyte CD45 expression and viability after 72 h storage. Treated neutrophils were also functional after 72 h, as measured using a high-precision microfluidic device. For research purposes, rapid neutrophil degradation strains experimental timelines, as the cells must be evaluated immediately. The approach described here represents a simple method to overcome logistical challenges for implementing functional neutrophil assays for research and clinical applications^2, 34^. While the focus of this study was on purified neutrophils, preliminary work shows this approach may also be effective for neutrophils in whole blood, though further work is necessary to determine whether this observation is reproducible among unique donors (Supplemental Figure S3).

One consequence of storage-induced damage is the transition of erythrocyte phenotype from discoid to echinocyte, and was therefore selected as a marker of deterioration^35^. Further, as echinocytes may interfere with microfluidic cell sorting, there are benefits to preserving discoid morphology (Supplemental Table S1). CS-original was shown to significantly increase the number of discoid erythrocytes in healthy donor whole blood, but was inadequate for preserving blood specimens from women with metastatic breast cancer, where erythrocytes were found to deteriorate at a more rapid rate than blood from healthy donors. This increased rate of deterioration could explain why CS-original was insufficient to stabilize patient samples, as vesicles can form at the tips of echinocyte spicules and bud off in a time progressive manner that is proportional to storage duration^36^. During rejuvenation, the echinocyte-discoid transition cannot be completed due to irreversible membrane loss^37, 38^. Such differences might result either from the effect of chemotherapeutic toxicity on blood cell precursors, or from history of blood transfusions, both of which are common in the patient population tested. Consequently, CS-original was reformulated to preserve patient blood using Taguchi methods of experimental design. The optimized formulation combined with Q-VD-OPh, CS_WB_, significantly improved leukocyte viability and erythrocyte morphology after 72 h ambient storage compared to untreated controls in blood from both healthy donors and women with metastatic breast cancer.

The data reported here demonstrate that CS_WB_ stabilizes erythrocyte morphology and leukocyte viability. However, there are many limitations to this study that must be addressed in order to establish the effectiveness of CS_WB_ as a true “whole blood” preservative. First, this study was limited in scope to women with metastatic breast cancer as a model of disease. Future studies will therefore evaluate CS_WB_ in other disease types and patient populations. Second, we limited our study to a manageable subset of descriptors to characterize blood preservation. This includes erythrocyte morphology and leukocyte viability in whole blood, and neutrophil chemotaxis and NETosis in purified cell suspensions. However, there are many additional descriptors that are important for both clinical and research applications. This includes further evaluation of neutrophil function (phagocytosis, respiratory burst, etc.) as well as functional characterization of other leukocyte subsets such as lymphocytes. Further evaluation is also necessary to determine whether erythrocyte function (oxygen delivery, deformability, etc.) is preserved. Platelets are another important component of blood. Preliminary studies demonstrate that CS_WB_ does not negatively impact platelet viability, though an increase in platelet activation was observed where 8.1 ± 7.2% of CS_WB_ platelets were activated compared to 2.8 ± 4.3% of fresh platelets (Supplemental Figure S4). Future studies will establish whether platelet function is altered during storage. These and additional descriptors will serve as a basis for more stringent criterion for preservation and will be the focus of future work.

The development of medical technologies that rely on peripheral blood for the diagnosis and prognosis of disease has created a need for preservatives that maintain blood in its viable state. While fixative-based blood preservatives are sufficient for some applications, storage solutions that enable recovery of viable cells are critical for emerging clinical and research areas such as cell culture, functional characterization of neutrophils, transfusions and molecular analysis. While there are several FDA approved preservatives for refrigerated storage of blood components, there has been little progress made on ambient whole blood storage. Short-term stabilization of whole blood would allow shipment of isolates between clinics or to centralized facilities and ease experimental timelines in research settings. CS_WB_ was shown here to stabilize erythrocyte morphology and leukocyte viability and CD45 biomarker expression for 72 h under ambient conditions. CS_WB_ may be particularly useful for emerging microfluidic diagnostic platforms capable of sorting blood cells based on their specific morphology and biomarker expression^39^. This time frame is sufficient to enable sample transport and addresses a significant bottleneck for the dissemination of emerging medical and research technologies that rely on viable blood cells. Because CS_WB_ was developed for ambient temperature storage, this preservative would be useful for low-resource settings where isolates often require transport from field to clinic or laboratory. In summary, CS_WB_ is affordable and simple to prepare and can be immediately implemented into existing research methods.

## Electronic supplementary material

Supplementary Information

Supplementary Video S1. Migration of Q-VD-OPh treated neutrophils following 96 h ambient storage.

Supplementary Video S2. Migration of untreated control neutrophils following 96 h ambient storage.

## References

[1] Snyder KB, Rgmathew AJ, Baust JG, Baust JM (2004). Biological packaging for the global cell and tissue therapy markets. BioProcessing Journal.

[2] Jones CN (2014). Spontaneous neutrophil migration patterns during sepsis after major burns. PLoS One.

[3] Yu M (2014). *Ex vivo* culture of circulating breast tumor cells for individualized testing of drug susceptibility. Science.

[4] Berendsen TA (2014). Supercooling enables long-term transplantation survival following 4 days of liver preservation. Nat Med.

[5] Ploeg RJ (1988). Successful 72-hour cold storage kidney preservation with UW solution. Transplant Proc.

[6] Collins GM, Bravo-Shugarman M, Terasaki PI (1969). Kidney preservation for transportation. Initial perfusion and 30 hours’ ice storage. Lancet.

[7] Greenbaum A, Hasany SM, Rootman D (2004). Optisol vs Dexsol as storage media for preservation of human corneal epithelium. Eye (Lond).

[8] Armitage WJ (2011). Preservation of Human Cornea. Transfus Med Hemother.

[9] Oh, H., Siano, B. & Diamond, S. Neutrophil isolation protocol. *J Vis Exp* (2008). 10.3791/745 PMC3074468 19066523

[10] Dumaswala UJ, Rugg N, Greenwalt TJ (1994). Studies in red blood cell preservation: 9. The role of glutamine in red cell preservation. Vox Sang.

[11] Wong KH (2016). The Role of Physical Stabilization in Whole Blood Preservation. Sci Rep.

[12] Blasi B, D’Alessandro A, Ramundo N, Zolla L (2012). Red blood cell storage and cell morphology. Transfus Med.

[13] Beech JP, Holm SH, Adolfsson K, Tegenfeldt JO (2012). Sorting cells by size, shape and deformability. Lab Chip.

[14] Högman CF, Hedlund K, Zetterström H (1978). Clinical usefulness of red cells preserved in protein-poor mediums. N Engl J Med.

[15] Hess JR (2006). An update on solutions for red cell storage. Vox Sanguinis.

[16] Heaton A (1984). Use of Adsol preservation solution for prolonged storage of low viscosity AS-1 red blood cells. Br J Haematol.

[17] Simon TL, Marcus CS, Myhre BA, Nelson EJ (1987). Effects of AS-3 nutrient-additive solution on 42 and 49 days of storage of red cells. Transfusion.

[18] Murphy S, Gardner FH (1969). Effect of storage temperature on maintenance of platelet viability–deleterious effect of refrigerated storage. N Engl J Med.

[19] Chaudhary R, Katharia R (2012). Oxidative injury as contributory factor for red cells storage lesion during twenty eight days of storage. Blood.

[20] Collard K, White D, Copplestone A (2014). The influence of storage age on iron status, oxidative stress and antioxidant protection in paediatric packed cell units. Blood Transfus.

[21] Mohanty JG, Nagababu E, Rifkind JM (2014). Red blood cell oxidative stress impairs oxygen delivery and induces red blood cell aging. Front Physiol.

[22] Stowell SR (2013). Addition of ascorbic acid solution to stored murine red blood cells increases posttransfusion recovery and decreases microparticles and alloimmunization. Transfusion.

[23] Pallotta V, Gevi F, D’alessandro A, Zolla L (2014). Storing red blood cells with vitamin C and N-acetylcysteine prevents oxidative stress-related lesions: a metabolomics overview. Blood Transfus.

[24] Rao RS, Kumar CG, Prakasham RS, Hobbs PJ (2008). The Taguchi methodology as a statistical tool for biotechnological applications: a critical appraisal. Biotechnol J.

[25] Taguchi, G. *System of Experimental Design: Engineering Methods to Optimize Quality and Minimize Costs*. U. K. I. Publications, Eds, (White Plains, N. Y., 1987), Vols 1 & 2.

[26] Boneschansker L, Yan J, Wong E, Briscoe DM, Irimia D (2014). Microfluidic platform for the quantitative analysis of leukocyte migration signatures. Nat Commun.

[27] Alhanaty E, Sheetz MP (1981). Control of the erythrocyte membrane shape: recovery from the effect of crenating agents. J Cell Biol.

[28] Lim G, Wortis M, Mukhopadhyay R (2002). Stomatocyte-discocyte-echinocyte sequence of the human red blood cell: Evidence for the bilayer-couple hypothesis from membrane mechanics. Proceedings of the National Academy of Sciences of the United States of America.

[29] Wardle DJ (2011). Effective caspase inhibition blocks neutrophil apoptosis and reveals Mcl-1 as both a regulator and a target of neutrophil caspase activation. PLoS One.

[30] Cowburn AS, White JF, Deighton J, Walmsley SR, Chilvers ER (2005). z-VAD-fmk augmentation of TNF alpha-stimulated neutrophil apoptosis is compound specific and does not involve the generation of reactive oxygen species. Blood.

[31] Caserta TM, Smith AN, Gultice AD, Reedy MA, Brown TL (2003). Q-VD-OPh, a broad spectrum caspase inhibitor with potent antiapoptotic properties. Apoptosis.

[32] Ch’en IL (2008). Antigen-mediated T cell expansion regulated by parallel pathways of death. Proc Natl Acad Sci USA.

[33] Degterev A (2005). Chemical inhibitor of nonapoptotic cell death with therapeutic potential for ischemic brain injury. Nat Chem Biol.

[34] Sackmann EK (2014). Characterizing asthma from a drop of blood using neutrophil chemotaxis. Proc Natl Acad Sci USA.

[35] Holme S (2005). Current issues related to the quality of stored RBCs. Transfus Apher Sci.

[36] Greenwalt TJ, Bryan DJ, Dumaswala UJ (1984). Erythrocyte membrane vesiculation and changes in membrane composition during storage in citrate-phosphate-dextrose-adenine-1. Vox Sang.

[37] Laczkó J, Szabolcs M, Jóna I (1985). Vesicle release from erythrocytes during storage and failure of rejuvenation to restore cell morphology. Haematologia (Budap).

[38] Flatt JF, Bawazir WM, Bruce LJ (2014). The involvement of cation leaks in the storage lesion of red blood cells. Front Physiol.

[39] Karabacak NM (2014). Microfluidic, marker-free isolation of circulating tumor cells from blood samples. Nat Protoc.

