# Supplementary material for: Preservative solution that stabilizes erythrocyte morphology and leukocyte viability under ambient conditions
Source: Sci Rep. 2017 Jul 18;7:5658. doi: 10.1038/s41598-017-05978-7 (PMC5515929; doi:10.1038/s41598-017-05978-7)
Supplement: Supplementary file 1 — Supplementary Information [file 41598_2017_5978_MOESM1_ESM.pdf]

Supplementary Information

Preservative solution that stabilizes erythrocyte morphology and leukocyte viability under ambient conditions

Rebecca D. Sandlin,[1] Keith H. K. Wong,[1] Leo Boneschansker,[1] Thomas R. Carey,[1] Kathleen L. Miller,[1] Gregory Rose,[1] Daniel A. Haber,[2,3] Shyamala Maheswaran,[4] Daniel Irimia,[1] Shannon L. Stott,[5,*] Mehmet Toner[1,*]

[1]BioMEMS Resource Center, Center for Engineering in Medicine, & Department of Surgery, Massachusetts General Hospital, Harvard Medical School, Boston, MA 02114, USA.

[2]Cancer Center & Department of Medicine, Massachusetts, MA General Hospital, Harvard Medical School, Boston, MA 02114, USA.

[3]Howard Hughes Medical Institute, Chevy Chase, MD 20815, USA.

[4]Cancer Center & Department of Surgery, Massachusetts General Hospital, Harvard Medical School, Boston, MA 02114, USA.

[5]Cancer Center, Department of Medicine, & BioMEMS Resource Center, Center for Engineering in Medicine, Massachusetts General Hospital, Harvard Medical School, Boston, MA 02114, USA.

[*]To whom correspondence should be addressed:

Shannon L. Stott, Email: SSTOTT@mgh.harvard.edu

Mehmet Toner, Email: mtoner@hms.harvard.edu

Image of product following microfluidic purification to remove erythrocytes from nucleated cells	#contaminating erythrocytes /µL product
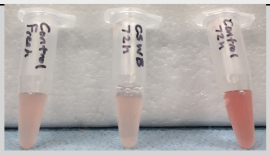<br>Fresh control · 72h, CS_{WB} treated · 72h, untreated control	Fresh blood (control): 6,800<br>72 h CS_{WB}-treated: 8,160<br>72 h, untreated control: 25,360
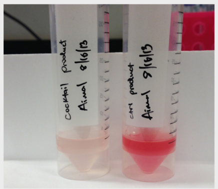<br>96h, CS-original treated · 96h, untreated control	Fresh control: N/A<br>96 h CS-original-treated: 1,500<br>96 h, untreated control: 21,750
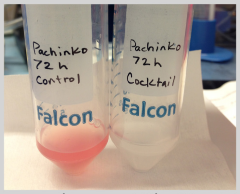<br>72h, untreated control · 72h, CS-original treated	Fresh control: N/A<br>72 h CS-original-treated: 470<br>72 h, untreated control: 10,000

Supplementary Table S1. Echinocyte formation interferes with microfluidic cell sorting. Compared to discoid erythrocytes, echinocytes exhibit an increased cell radius along the minor axis, which may interfere with microfluidic cell sorting for blood cell purification. This observation was previously reported using deterministic lateral displacement (DLD) to sort cells by size and shape.(*1*) Here, we show examples using the CTC iChip, a microfluidic device that removes erythrocytes from nucleated cells by DLD. (*2, 3*) The CTC iChip is capable of purifying circulating tumor cells (CTC) from peripheral blood. In the examples shown here, blood was stored for 72-96 h. A higher percentage of echinocytes was observed in the untreated controls compared to CS-original or CS_{WB} treated blood. Consequently, the product of untreated controls contains more erythrocyte contamination compared to the CS-original or CS_{WB}-treated samples due to less efficient cell sorting.

Study ID	Age at primary diagnosis	Stage at primary diagnosis	Type of breast cancer at primary diagnosis (ductal, lobular, mixed, other)	Age at metastatic diagnosis	ER status	PR status	HER2 status	Breast Cancer subtype	Age at death (if applcable)
BRx-107	53	IIA	Ductal	55	Positive	Positive	Negative	HR+/HER2-	60
BRx-222	52	Unk	Unk	80	Positive (faintly)	Positive	Negative	HR+/HER2-	N/A
BRx-145	44	Unk	Ductal	70	Positive	Positive	Negative	HR+/HER2-	N/A
BRx-175	39	I	Mixed	54	Positive	Positive	Negative	HR+/HER2-	N/A
BRx-210	53	IIIA	Ductal	54	Positive	Positive	Negative	HR+/HER2-	N/A
BRx-179	49	IIB	Ductal (focal lobular neoplasia)	62	Positive	Positive	Negative	HR+/HER2-	N/A

Supplementary Table S2. Clinical details on patients selected for validation of CS_{WB}.

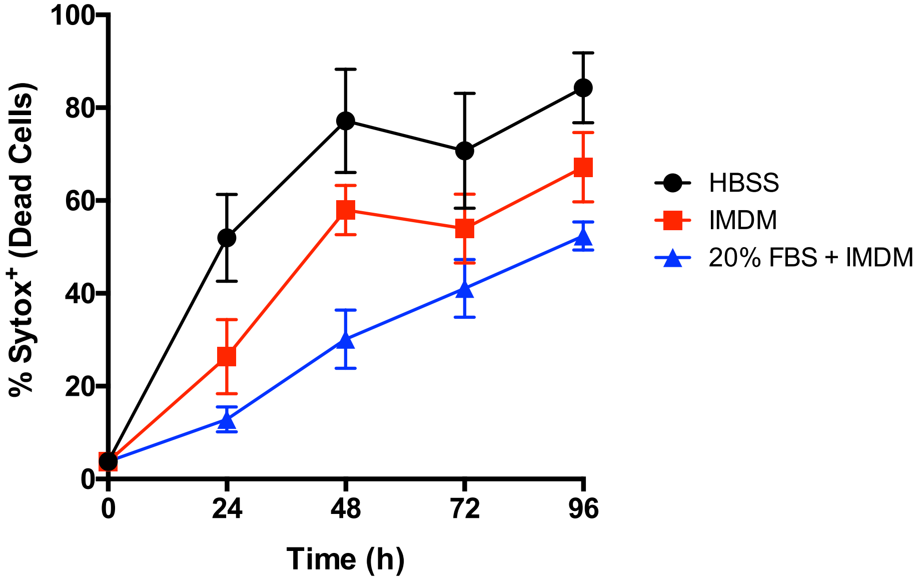

Supplementary Figure S1. Identification of base media conditions for neutrophil storage. To determine the optimal base media for preserving isolated neutrophils under ambient conditions, we examined the effects of HBSS, IMDM and 20%FBS/IMDM (n=3 independent donors, mean ± standard deviation). Dead cells were quantified using flow cytometry and SYTOX Green positivity.

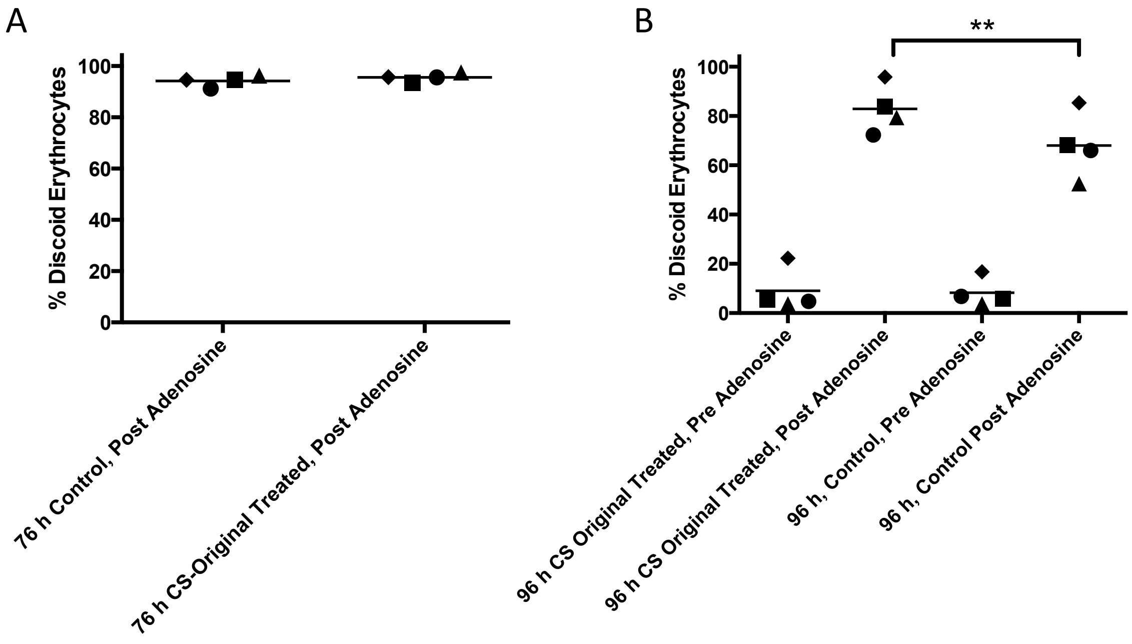

Supplementary Figure S2. CS-Original stabilizes healthy donor blood. Blood was obtained from four independent healthy donors, divided into 1 mL samples and treated with CS-Original or untreated (whole blood control). (A) Following 72 h ambient storage, blood was treated with adenosine at 37°C for 4h to rejuvenate erythrocytes (see methods). Erythrocyte morphology was then manually assessed. Following the adenosine treatment (76 h), 94.2 ± 2.1% of erythrocytes in the CS-Original treated samples exhibited discoid morphology compared to 95.6 ± 1.6% in the control. (B) The same protocol was repeated for blood stored under ambient conditions fro 96 h. Prior to the adenosine treatment, erythrocytes exhibited significant deterioration, though improvement was observed following the adenosine rejuvenation treatment where 82.9 ± 9.9% of erythrocytes in the CS-Original treated samples exhibited discoid morphology compared to 68.1 ± 13.5% in the control (mean ± standard deviation, ***p* < 0.01, 1-way repeated measures ANOVA).

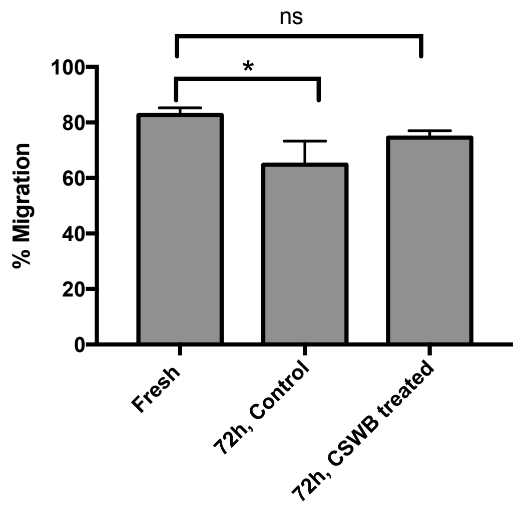

Supplementary Figure S3. Evaluation of neutrophil function following 72 h storage in whole blood. A preliminary experiment was completed to determine whether CS_{WB}-treated neutrophils stored in whole blood maintain function following 72 h ambient storage. Peripheral blood obtained from a healthy donor was immediately aliquoted into three falcon tubes (5 mL each for fresh control, 72 h control and 72 h CS_{WB}-treated). Neutrophils were immediately isolated from the fresh control using the EasySep Human Neutrophil Enrichment Kit (STEMCELL Technologies, Vancouver, Canada) using the manufacturer's protocol. Neutrophils were then characterized using a microfluidic chemotaxis device (see methods).(*4*) Following 72 h ambient storage, neutrophil isolation and characterization was performed in an identical manner to the fresh control. In the fresh control, 82.7 ± 2.6% of the neutrophils migrated compared to 64.7 ± 8.5% and 74.5 ± 2.5% in the 72 h untreated control and 72 h CS_{WB}-treated sample, respectively. The migration data obtained from this experiment suggest that CS_{WB} confers improvement in neutrophil chemotaxis following storage compared to the untreated control. Future studies will focus on whether this observation is reproducible among unique donors (data represents n=1 healthy donor, plotted as mean ± SEM for technical replicates, n=6 fresh, n=3 72 h control, n=3 72 h CS_{WB} treated, $P<0.05$, t test).

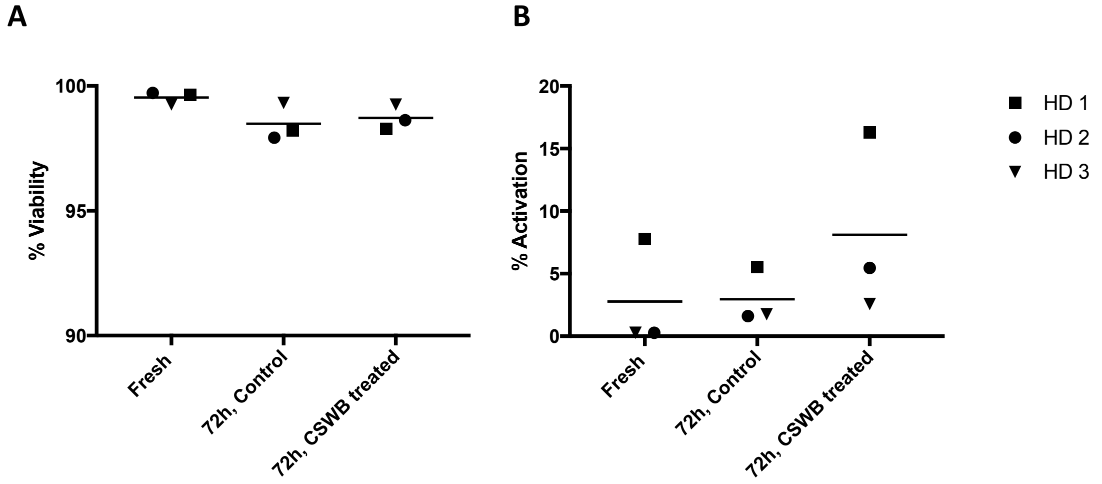

Supplementary Figure S4. Platelet viability and activation following ambient storage. Blood from three unique healthy donors was obtained and stored under ambient conditions to determine whether CS_{WB} affects platelet viability or activation. Following the indicated time interval, blood was diluted in PBS (1:60) and stained with CD41 Pacific Blue platelet biomarker (1:150), CD62p platelet activation biomarker (1:100) and calcein viability stain (0.2 µM) and analyzed using an ImageStream^{X} Mk. II Imaging Flow Cytometer (Amnis Corporation). 30,000 events were collected for each sample. Data analysis was performed using the IDEAS software package. Focused, single, CD41 positive events were gated from the cell population. Gates were applied to determine CD62p and calcein intensity. (A) Viability remained at >95% for all samples, regardless of treatment. (B) For CS_{WB}-treated blood, a slight increase in platelet activation was observed. Additional studies are necessary to determine whether platelets stored in CS_{WB} maintain functional integrity.

Supplementary Video S1. Migration of Q-VD-OPh treated neutrophils following 96 h ambient storage. Purified neutrophils were treated with Q-VD-OPh and stored under ambient conditions for 96 h. Neutrophil migration was then analyzed using a previously described microfluidic device.(*4*) Neutrophils were introduced into the main channel (located in the center of the video). The main channel is connected to a reservoir containing fMLP (100 nM). Sensing the chemical gradient, the neutrophils can be seen migrating toward this reservoir.

Supplementary Video S2. Migration of untreated control neutrophils following 96 h ambient storage. Purified neutrophils were stored under ambient conditions as described. After 96 h, neutrophils were loaded into a microfluidic device as describe in Video 1. It is evident that neutrophils treated with Q-VD-OPh undergo a greater degree of migration compared to untreated controls.

References

1. J. P. Beech, S. H. Holm, K. Adolfsson, J. O. Tegenfeldt, Sorting cells by size, shape and deformability. *Lab Chip* **12**, 1048-1051 (2012).
2. N. M. Karabacak *et al.*, Microfluidic, marker-free isolation of circulating tumor cells from blood samples. *Nat Protoc* **9**, 694-710 (2014).
3. E. Ozkumur *et al.*, Inertial Focusing for Tumor Antigen-Dependent and -Independent Sorting of Rare Circulating Tumor Cells. *Science Translational Medicine* **5**, 179ra147 (2013).
4. L. Boneschansker, J. Yan, E. Wong, D. M. Briscoe, D. Irimia, Microfluidic platform for the quantitative analysis of leukocyte migration signatures. *Nat Commun* **5**, 4787 (2014).
